# Canga biodiversity, a matter of mining

**DOI:** 10.3389/fpls.2014.00653

**Published:** 2014-11-24

**Authors:** Aleksandra Skirycz, Alexandre Castilho, Cristian Chaparro, Nelson Carvalho, George Tzotzos, Jose O. Siqueira

**Affiliations:** ^1^Department of Sustainable Development, Vale Institute of TechnologyBelém, Brazil; ^2^Vale S.A., AP Supervisao PCMCarajas, Brazil

**Keywords:** canga, iron, ecosystem, endemism, restoration

## Abstract

Brazilian name canga refers to the ecosystems associated with superficial iron crusts typical for the Brazilian state of Minas Gerais (MG) and some parts of Amazon (Flona de Carajas). Iron stone is associated with mountain plateaux and so, in addition to high metal concentrations (particularly iron and manganese), canga ecosystems, as other rock outcrops, are characterized by isolation and environmental harshness. Canga inselbergs, all together, occupy no more than 200 km^2^ of area spread over thousands of km^2^ of the Iron Quadrangle (MG) and the Flona de Carajas, resulting in considerable beta biodiversity. Moreover, the presence of different microhabitats within the iron crust is associated with high alpha biodiversity. Hundreds of angiosperm species have been reported so far across remote canga inselbergs and different micro-habitats. Among these are endemics such as the cactus *Arthrocereus glaziovii* and the medicinal plant *Pilocarpus microphyllus.* Canga is also home to iron and manganese metallophytes; species that evolved to tolerate high metal concentrations. These are particularly interesting to study metal homeostasis as both iron and manganese are essential plant micro-elements. Besides being models for metal metabolism, metallophytes can be used for bio-remediation of metal contaminated sites, and as such are considered among priority species for canga restoration. “Biodiversity mining” is not the only mining business attracted to canga. Open cast iron mining generates as much as 5–6% of Brazilian gross domestic product and dialog between mining companies, government, society, and ecologists, enforced by legal regulation, is ongoing to find compromise for canga protection, and where mining is unavoidable for ecosystem restoration. Environmental factors that shaped canga vegetation, canga biodiversity, physiological mechanisms to play a role, and ways to protect and restore canga will be reviewed.

## CANGA AND SIMILAR ECOSYSTEMS WORLD-WIDE

**FIGURE 1:**
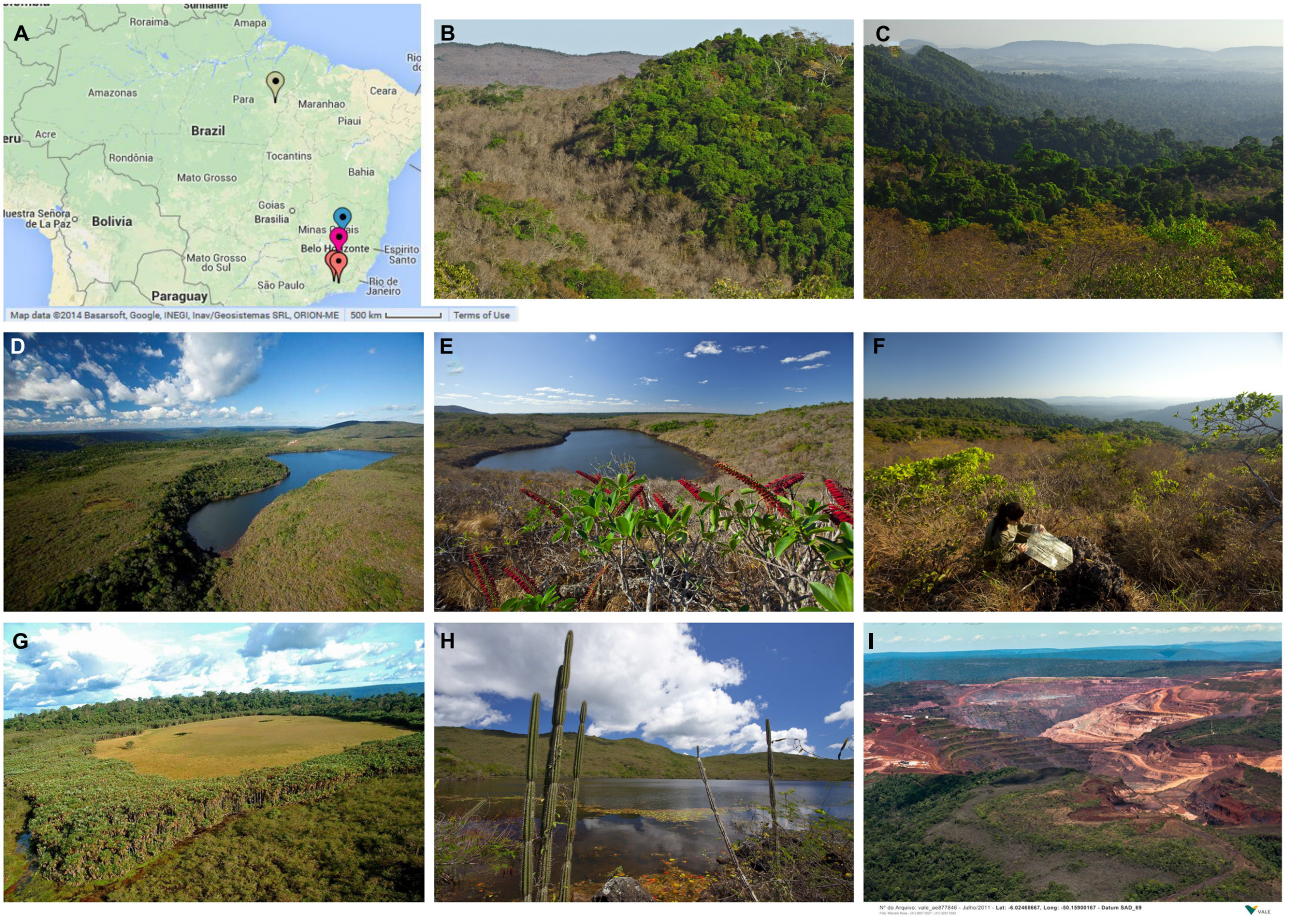
**Canga (A) Location of canga inselbergs investigated in Carajas and IQ.** Map generated and reproduced from Google Maps. Green indicator (Nunes, 2009), red indicators (Jacobietal., 2007), violet indicator (Viana and Lombardi, 2007); blue indicator (Pifano etal., 2010). **(B–H)** Photographs of canga taken in Crajas South Range, courtesy of Alexandre Castilho. **(I)** Mining operation in the Carajas region, courtesy of Alexandre Castilho.

In Brazil term canga refers to the ecosystem associated with superficial iron crust and present in the states of Minas Gerais (MG; Quadrilatero Ferrifero) and Para (Serra de Carajas; **Figure 1A**; **Table 1**). Literally translated MG stands for “general mines” reflecting strong ties to the mining industry due to its rich mineral deposits. The iron quadrangle (IQ) covers approximately 7200 km^2^ with the superficial iron crusts distributed on the tops of mountains at altitudes ranging from 1000 to 2000 m above the sea level. The total area covered by canga in the IQ is relatively small, approximately 100 km^2^ (Dorr, 1964), and dispersed on mountain inselbergs (600–700 m above the sea level). Similarly, in the Amazon state of Para (**Figure 1A**) canga occupies only 2 or 3% of natural clearings on iron rocks of the Carajas mountains, approximately 90 km^2^, with the surrounding area covered by dense tropical forest (Nunes, 2009).

**Table 1:** Comparison of iron based ecosystems in Brasil (canga) and Australia (bounded iron formations).

	Canga in IQ	Canga in Carajas	BIFs
Location	Minais Gerais (MG), Brazil	Para, Brazil (Nunes, 2009)	South-Western Australia
*Longitude*	19.30, 20.31 S (Jacobi et al., 2007)	5.35, 5.57 S (Nunes, 2009)	25.30, 30.00 S (Gibson et al., 2012)
*Latidude*	43.00-44.30 W (Jacobi et al., 2007)	50.01, 51.04 W (Nunes, 2009)	116.00, 122.15 E (Gibson et al., 2012)
Approximate area	100km^2^ (Jacobi et al., 2007)	90 km^2^ (Nunes, 2009)	–
Spread across area/range	Area of 7200km^2^ (Jacobi et al., 2007)	Area of 4000 km^2^ (Nunes, 2009)	Across 700 km (Gibson et al., 2012 )
Altidue	900–2000 m (Jacobi et al., 2007)	Max 620–660 m	Max 700 m (Gibson et al., 2012)
Climate	Tropical sub-humid (Jacobi et al., 2007)	Tropical humid (Nunes, 2009)	Arid (Gibson et al., 2012)
*Minimum temperature*	13–19∘C	19∘C (Nunes, 2009)	3.8–7.5∘C (Gibson et al., 2012)
*Maximum temperature*	24–28∘C	31∘C (Nunes, 2009)	34-39∘C (Gibson et al., 2012)
*Mean precipitation*	1500–1900 mm (Jacobi et al., 2007)	1900 mm (Nunes, 2009)	186–329 mm (Gibson et al., 2012)
*Rain seasonality*	Yes (Jacobi et al., 2007)	Yes (Nunes, 2009)	Yes (Gibson et al., 2012)
Soil characteristics*	Messias et al. (2013)	Nunes (2009)	Markey and Dillon (2010)
*pH*	4.7–5.15 (Messias et al., 2013)	3.76–4.37 (Nunes, 2009)	4.22–5.97 (Markey and Dillon, 2010)
*Mn (mg/kg)*	10 times higher (Messias et al., 2013)	Two times higher (Nunes, 2009)	4–20 times higher (Markey and Dillon, 2010)
*Fe (mg/kg)*	20–40 times higher (Messias et al., 2013)	20–50 times higher (Nunes, 2009)	2–6 times higher (Markey and Dillon, 2010)
*P(mg/kg)*	10–20 times lower (Messias et al., 2013)	5–20 times lower (Nunes, 2009)	10 times lower (Markey and Dillon, 2010)
Surrounding ecosystem	Atlantic rain-forest, Cerrado, rock outcrops (Jacobi et al., 2007)	Amazonian rain-forest (Nunes, 2009)	Arid and Transition Rainfall Zones (Gibson et al., 2012)
Plant Biodiversity***	Jacobi et al. (2007), Viana and Lombardi (2007), Pifano et al. (2010)	Nunes (2009)	Gibson et al. (2012)
*Number of plant species*	353 (Jacobi and Carmo, 2011)	125 (Nunes, 2009)	906 (Gibson et al., 2012)
*Dominant genus 1*	Asteraceae (Jacobi and Carmo, 2011)	Fabaceae (Nunes, 2009)	Fabaceae (Gibson et al., 2012)
*Dominant genus 2*	Poaceae (Jacobi and Carmo, 2011)	Euphorbiaceae (Nunes, 2009)	Myrtaceae (Gibson et al., 2012)
*Dominant genus 3*	Orchidaceae (Jacobi and Carmo, 2011)	Myrtraceae (Nunes, 2009)	Poaceae (Gibson et al., 2012)
*Dominant life form 1*	Herbs (Jacobi and Carmo, 2011)	Shrubs (Nunes, 2009)	Shrubs (Gibson et al., 2012)
*Dominant life form 2*	Shrubs (Jacobi and Carmo, 2011)	Herbs (Nunes, 2009)	Herbs (Gibson et al., 2012)
Presence of endemics	Yes (Jacobi et al., 2007)	Yes (Nunes, 2009)	Yes (Gibson et al., 2012)

*Aproximate diffrences in element composition when compared to standard soil (Reganold et al., 2010). **Dominance calculated based on number of species.

Canga-like areas, characterized by the presence of ironstone, ferricrete soils, are not restricted to Brazil; however, in other parts of the world they would be recognized under a different name. Canga-like habitats, refered to as banded iron formations (BIFs) have been reported in South-Western Australia (Gibson et al., 2010, 2012; **Table 1**). Similarly to canga, BIFs form isolated inselbergs (some 700 km apart) on tops of mountains and are characterized by high local and regional diversity. As canga they are endangered by the mining industry and their restoration provides a major challenge (Gibson et al., 2010).

## ENVIRONMENTAL FORCES THAT SHAPED CANGA BIODIVERSITY

The canga ecosystem has been shaped by 1000s of years long evolution process that gave rise to communities thriving in unique and severe environmental conditions. As is the case with other mountain top outcrops, the canga biome had to adapt to high ultraviolet (UV) exposure, high daily temperatures, rapid water loss, strong winds and poorly developed soil cover (Jacobi et al., 2007). High transpiration and low soil retention capacity challenges plants during periods of drought, even during the wet season. In addition to canga soils being shallow, acid (pH∼4.0, in comparison to pH∼7.0 of for the optimal agricultural soils) and nutritionally poor, they also contain toxic levels of aluminum and heavy metals (**Table 1**). More specifically canga substrates contain little phosphorous (P), magnesium (Mg), and calcium (Ca) but plenty of iron (Fe) and manganese (Mn; Vincent and Meguro, 2008; Oliveira et al., 2009; Silva, 2013). Copper (Cu), nickel (Ni), zinc (Zn), chromium (Cr), and lead (Pb) accumulation has been also reported in plant samples harvested from the canga outcrop (Porto and da Silva, 1989; Silva, 1992). Importantly metal toxicity is aggravated by low pH, which increases metal mobility and so accessibility for plant uptake, e.g., under low pH iron is released from otherwise insoluble ferric oxides (Morrissey and Guerinot, 2009).

Adding to the severe abiotic conditions, the floral composition of canga is also a result of mineral and topographical heterogeneity giving rise to distinct microhabitats within canga (e.g., Jacobi et al., 2007; Viana and Lombardi, 2007). Iron-rich substrates may be totally fragmented or form a thick, solid crust. Cliffs, caves, grassland, rock fields, cracks in rock crevices, depressions, temporary or permanent ponds, temporally flooded areas all are part of canga. Finally canga biodiversity is affected by the surrounding ecosystems (Jacobi and Carmo, 2008a); in Carajas canga constitutes an enclave within an Amazonian forest whilst in IQ canga is located in the transition zone between the Cerrado and Atlantic Forest.

To summarize, harsh environmental conditions have supported evolution of hardy and often unique plants that can thrive in dry, metal rich but nutrient poor soil. High biodiversity is a result of canga being not one but rather a conglomerate of distinct microhabitats. It is dispersed along mountain tops rather than being a continuous habitat, and high biodiversity of the surrounding ecosystems.

## PLANT BIODIVERSITY PORTFOLIO

Canga is home to 100s of plant species (Jacobi and Carmo, 2011). Close to 500 have been reported so far, sampled from only a small percentage of the total area occupied by canga. Three taxonomic studies published for the IQ (Jacobi et al., 2007; Viana and Lombardi, 2007; Pifano et al., 2010) and one for the Carajas Mountains (Nunes, 2009) will be referred to here.

Looking at plant species several characteristics emerge. Firstly, canga floristic composition significantly differs from the neighboring ecosystems. When compared with the Atlantic and Amazonian forest (Nunes, 2009; Pifano et al., 2010) the difference is obvious at first sight; canga landscape resembling savanna covered by shrubs and herbaceous plants with few trees and associated crawlers (**Figures 1B–H**). Thus not surprisingly, 80% of species identified in canga was also found to be canga exclusive in comparison with the Atlantic forest, swamp and riparian forest (Pifano et al., 2010). Less obviously, the canga floristic composition also differs from other rocky outcrops present in IQ. A comparison of the 16 most represented angiosperm families found in sandstone and canga rocky outcrops revealed significant differences. For instance, whereas Solanaceae and Verbenaceae species are absent in the sandstone inselbergs they are abundant in canga. The opposite being true for Eriocaulacea and Xyridaceae angiosperms (Jacobi and Carmo, 2008b).

Secondly, canga is characterized by high beta diversity, which refers to the differences measured between distinct canga inselbergs (Jacobi et al., 2007). In their work Jacobi et al. (2007) investigated two sites approximately 32 km apart. Out of 235 fern and angiosperm species only 27% were common for both sites. Even less overlap is found between the three studies from IQ, cangas 100s of kilometers apart. Only two species were identified in all studies and 18% were shared between any of the two. And so not surprisingly, the plant composition in the Carajas mountains, 1000 km away, shares only seven species common with those found in the IQ.

Thirdly, canga is characterized by high alpha biodiversity, which refers to differences measured withing a single canga inselberg (Jacobi et al., 2007). High alpha biodiversity is strongly related to the presence of distinct microhabitats within canga; differences in the amount and composition of soil, and moisture being considered the main factors shaping diverse plant communities (Vincent and Meguro, 2008; Nunes, 2009; Silva, 2013). A comprehensive study of Viana and Lombardi (2007) reported 358 plant species representing 70 angiosperm plant families, which is approximately 15% of all known angiosperm families world-wide. Researchers examined and described four distinctive canga microhabitats. The majority of the plant species were found on so-called “grassy fields,” which as the name implies, form homogenous, in appearance, sea of grasses and sages mixed with subshrubs and shrubs, and growing on a fragmented iron crust. In contrast “rocky fields” represent more an alpine like ecosystem characterized by the diversity of perennial and annual herbs (both monocots and dicots) growing in rock crevices formed on a solid iron crust. Noteworthy, endemic species: cactus *Arthrocereus glaziovii*, bromeliads *Consimilis dyckia* and *Vriesea minarum*, orchid *Oncidium gracile*, *Sinningia rupicola*, and legume trees *Mimosa calodendron* are all found in the open “grassy” or “rocky” fields (Jacobi and Carmo, 2011). In places with deeper substrate and more organic matter forest islands are established with small tree species and shrubs covered with crawlers and epiphytes. Last microhabitat represents an area disturbed by human activity and is mostly similar to the grassy fields albeit with only one fourth of the species richness. Remarkably more than 60% of the plant species (235 out of 358) were unique to the given microhabitat and only five were present in all of them. This kind of microhabitat specificity was also reported by Jacobi et al. (2007) and Nunes (2009). Examples of other microhabitats found in canga and described by Jacobi et al. (2007) include small permanent rock pools inhabited by unicellular and filamentous algae, ponds formed during the wet season in shallow depressions and covered with rare monocotyledonous plants from the Eriocaulon genus, entrances of small caves underneath iron crust providing shade and humidity for mosses and delicate herbs.

Finally, it was demonstrated that the exact floristic composition of canga could vary between dry and wet seasons (Nunes, 2009; Silva, 2013). However, canga is home to relatively few annual plants (Jacobi and Carmo, 2011), partial or complete loss of aboveground organs is a common adaptation to dry season (Jacobi et al., 2007).

To our knowledge and to date, canga diversity was estimated exclusively based on taxonomic identification, which may lead to both under- or over-estimation (Frankham, 2010), the first being more common. To illustrate the point, suffice to mention a recent work of Nistelberger et al. (2014). Analysis of genomic and mitochondrial regions of the millipede species from the Australian BIFs pointed to the long-term isolation of distinctive populations, inhabiting remote inselbergs, and so the need to place each population into a separate conservation unit.

When looking at individual species canga biodiversity may seem overwhelming. However, there is uniformity when looking at vegetation type and plant families. In general canga vegetation can be described as rocky, ephilitic (growing on stone), shrub dominated with large number of sedges, grasses, and orchids. Plant species are evenly distributed between shrubs, subshrubs, herbs, and epiphytes. Trees and crawlers are by far less diverse (Jacobi and Carmo, 2011). In terms of families the richest are Poaceae (grasses and sedges), Asteraceae (daisy like herbs), Fabaceae (legumes), Myrtaceae (subshrubs and shrubs), Melastomataceae (shrubs and small trees), and Orchidaceae (orchids; Jacobi et al., 2007; Viana and Lombardi, 2007; Nunes, 2009; Pifano et al., 2010). It is, however, important to mention that these families are not only rich in canga but also in other rocky outcrops (Jacobi and Carmo, 2008b), and they encompass 35% of the total ∼31000 angiosperm species reported in Brazil (Forzza, 2010). Area-wise ironstone outcrops are dominated by shrubs, mainly dicots. Monocots register a large proportion of herbaceous plants (grasses and sedges) and subshrubs. Dicot herbs are represented by numerous species of relatively small population sizes (Jacobi et al., 2007). This general characteristic of canga outcrop is also shared with plant communities found in Australian BIFs (**Table 1**); BIFs landscape being dominated by shrubs and herbaceous species from the Fabaceae, Myrtaceae, and Poaceae families (**Table 1**), generously represented in canga (Gibson et al., 2012).

Summarizing, canga ecosystems are characterized by high local and regional diversity. To date, 100s of plant species, some endemic, have been identified across diverse canga microhabitats and geographical locations. The total area of canga is estimated to be approximately 200 km^2^, of which approximately 1–2% was studied, pointing the need of additional investigation. Genomics tools can play a crucial role for the accurate estimation of genetic diversity.

## CANGA AS SOURCE OF NEW METAL HYPER-ACCUMULATING SPECIES

To cope with the harsh environmental conditions canga plants developed a number of physiological adaptations, of which metal tolerance is by far the most interesting. Together with Australian BIFs, cangas may constitute one of few ecosystems worldwide to “mine” for plants adapted to high iron. We are reviewing the importance of this in this section.

Metallophytes, that is species that evolved on metalliferous soils, are commonly divided into those that can exclude metals and those that can accumulate or even hyper-accumulate metals in their shoots (Baker, 1981; reviewed by Krämer, 2010 and Leitenmaier and Küpper, 2013). The first class represents majority of the species (>95%) that evolved avoidance mechanisms to block metal uptake into cells or mediate metal eﬄux and storage into root vacuoles, far from the photosynthetic cells. In contrast few percentages of plants would undergo extensive adaptation that would allow them to accumulate one or few metals in their shoots, where they act as repellent for herbivores and pathogens (Boyd, 2012). Metal transport from soil into plant shoot, complexation with organic compounds to reduce toxicity and sequestration inside vacuoles are the key processes considered for metal accumulation and tolerance (Krämer, 2010).

Growing on metal rich soils canga plant community is considered a unique source “to mine” for novel metal hyper-accumulating taxa. Of these manganese and iron hyper-accumulators stand out for a number of reasons. (1) In contrast to several 100 known nickel, cadmium, and zinc hyper-accumulators, not more than 20 manganese hyper-accumulating species were reported to date (Fernando et al., 2013). To our knowledge no iron accumulating taxa have been reported and studied. (2) Manganese and iron are essential plant micro-nutrients and their deficiency impacts plant yield (Marschner, 2012). But in high concentrations, manganese and iron are toxic to plants (Lynch and St Clair, 2004). As the unraveling molecular mechanisms underlying plant iron and manganese homeostasis is considered key to address both deficiency and toxicity issues reported for the agricultural crops, hyper-accumulators can be considered a good tool. For instance, analogous to zinc hyper-accumulators (Krämer, 2010), canga metallophytes are expected to encompass metal sensitive populations, which grow in ironstone outcrops along canga but in metal free substrate. Metal tolerant and metal sensitive populations within a species provide a great resource for genetic and molecular mining. On the one hand, recombinant inbred lines derived from parents differing in the trait of interest are the most common way to discover underlying QTLs. On the other hand, in system biology “omics” based analysis, differences in transcripts, proteins, and/or metabolites measured between tolerant and sensitive plants are good suspects behind observed phenotypes. (3) Soils with toxic levels of manganese are associated with mineral deposits but also with iron ore mining (strip mining generates manganese rich dust) and processing (steel industry uses big amounts of manganese). High concentration of manganese is toxic to plants but also to animals; in humans manganese poisons the nervous system resulting in Parkinson like symptoms (Taba, 2013). Because hyper-accumulators have the capacity to remove manganese from the soil they are considered a useful resource for phyto-remediation of post-industrial and post-mining areas (Whiting et al., 2004). (4) Finally, as metallophytes, similarly to endemics, are unique to canga they are a priority for post-mining canga restoration (see below).

**FIGURE 2:**
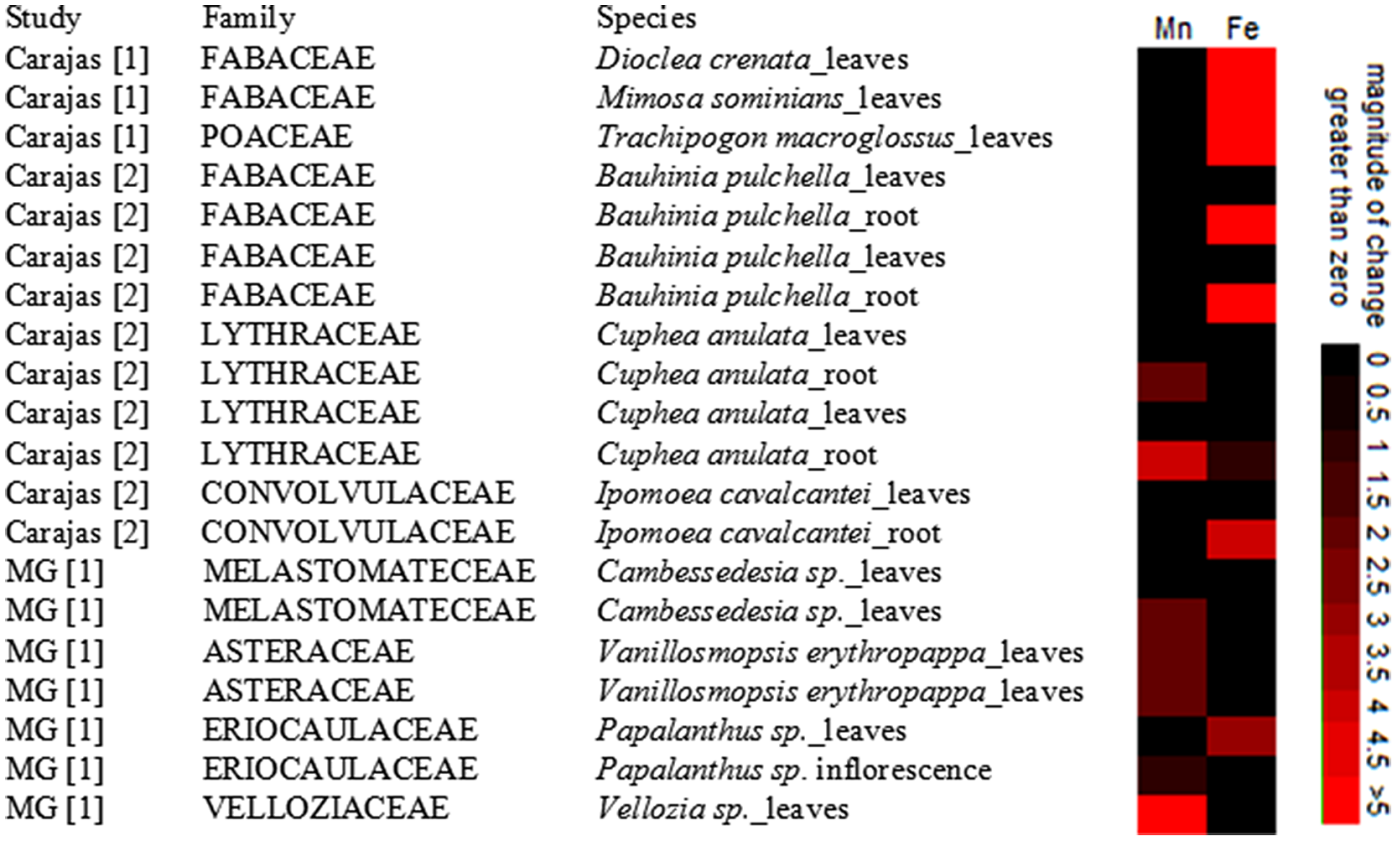
**Canga plant species accumulating at least double the standard concentration of manganese (200 ppm) and iron (300 pm).** From Porto and da Silva (1989) and Silva (1992). Two entries given to one specie indicate populations collected in different area of occurrence.

Despite the obvious interest, we found only two rather old studies reporting metal content of canga plants. Porto and da Silva (1989) and Silva (1992) examined 24 canga plant species, both from IQ and Carajas, for foliar concentration of Mn, Ni, Cr, Cu, Pb, and Fe. Twenty of the investigated specimens accumulated single or several metals in levels several times higher than “standard reference plant.” Although this is not enough to classify them as a hyper-accumulators (van der Ent et al., 2013) it is a good start for further canga research. Most interestingly, seven of the examined species accumulated at least double the iron or manganese leaf concentration of standard reference plants (**Figure 2**). Two more species accumulated iron and one more manganese in roots but not in their leaves (**Figure 2**). Concluding, studies of Porto and da Silva (1989) and Silva (1992) demonstrate that canga plants have developed various strategies to cope with high iron and manganese concentrations, through exclusion, or through accumulation, providing justification for additional studies.

To summarize quoting Krämer (2010): “metal hyper-accumulators will be instrumental in the development of systems biology approaches toward an integrated understanding of plant metal homeostasis” and Erskine et al. (2013): “to prevent continued biodiversity loss and to benefit from the unique adaptive mechanisms that exclude, tolerate, and even accumulate toxic metals in mine site rehabilitation, metallophytes must be recognized as vital asset at developmental stage of a mining operation.” From this perspective identification of canga metallophytes appears an obvious direction to pursue.

## OTHER PHYSIOLOGICAL ADAPTATION OF THE CANGA PLANTS

Besides metal tolerance and similar to the Australian BIFs (**Table 1**), canga plant community is known for its overall resilience to water scarcity (Jacobi et al., 2007). Rocky plant communities found in IQ are home to largest group of resurrection plants in Brazil, being able to tolerate almost complete desiccation by partial or complete loss of their above-ground organs during periods of severe drought. Less drastic adaptations include thick, waxy, imbricate leaves; guard cells sunken into pits; extensive and deep root systems; presence of water storing organs, e.g., pseudo-bulbs in orchids. Some plants, such as Clusa trees, are characterized by CAM photosynthesis, which allow them to keep guard cells shut during the day, by using night acquired CO_2_ stored in the organic acid malate. Slow growth rates of the canga plant species may be seen as yet another adaptation to water shortage. In stressful environments plants accumulate rather than spend their resources (carbon and water) in expense of growth, to avoid starvation if the conditions get worse (Skirycz et al., 2011).

## CANGA, METABOLITES, AND MEDICINAL PLANTS

To our knowledge, there is not an adequate molecular understanding of the physio-morphological traits that allow canga plants to thrive in their stressful environment. Of course parallels can be made from model plants but particulars need investigation. The potential of interesting findings can be demonstrated by available metabolite data. Many of the IQ plant species, present also in canga, were investigated for their metabolite profiles. These plants were those with reported medicinal properties and metabolite analysis was performed to investigate plausible agents responsible for the observed therapeutic affects (**Table 2**). Although these studies did not aim at understanding plant physiology, they revealed a richness of secondary metabolites associated with IQ ecosystem, with some of the compounds being well known for their involvement in plant stress responses. For example flavonols play a role in oxidative stress associated with drought and high UV (Skirycz et al., 2007) and past findings point to role of anthocyanin in metal complexion (Hale et al., 2001). Based on existing data, we believe that more comprehensive metabolite profiling of the canga plant species will shed light on the environmental adaptation of this unique rocky outcrop.

**Table 2:** Examples of secondary metabolites found in plant species from Minas Gerais (MG) and identified in canga outcrop by Jacobi et al. (2007).

Metabolite class	Example of compounds	Plant species	Reference
Terpenoids	Thymol, myrcene, cymene, terpinene, parthenin, beta-bisabolene	*Lippia gracilis, Lantana camara, cabralea canjerana, Copaifera langsdorffii, Lychnophora pinaster, Eremanthus erythropappus*	Guzzo et al. (2008), Sarria et al. (2011), Souza et al. (2011), Abreu et al. (2013), de Melo et al. (2013), Ferraz et al. (2013), Pradeep et al. (2013), Silvério et al. (2013), Zimmermam-Franco et al. (2013)
Phenylpropanoids Flavonoids	Catechin, methoxyflavanones, safrol	*Myrsine umbellata, Marcetia taxifolia, Tripodanthus acutifolius, Trixis vauthieri, Lychnophora pinaster, Ocotea cf.pulchella*	Ribeiro et al. (1997), Guzzo et al. (2008), Soberón et al. (2010), Leite et al. (2012), Abreu et al. (2013), Novaes et al. (2013), Leporatti et al. (2014)
Alkaloids		*Psychotria vellosiana*	Moraes et al. (2011)
Carotenoids	Lutein	*Myrsine umbellata*	Novaes et al. (2013)
Others	8-Methoxylapachenol, steroids, grifolin, grifolic acid, piperogalin	*Sinningia allagophylla, Lychnophora pinaster, Peperomia gallioides*	Langfield et al. (2004), Guzzo et al. (2008), Abreu et al. (2013), Barbosa et al. (2013)

Touching upon medicinal plants, it is interesting to mention the small tree *Pilocarpus microphyllus* (popular name jaborandi) endemic to Brazil and found in Flona de Carajas, where its associated with forest clearings and poor soils such as those present in canga. Leaves of Pilocarpus are collected by local communities and sold to pharmaceutical companies as source of the FDA approved anti-glaucome drug Timpilo (Costa, 2012). The main constituent of Timpilo is pilocarpine, an imidazole alkaloid inaccessible for cheap organic synthesis. To avoid irreversible depletion of the wild population, pilocarpine collection is regulated by the environmental agencies. Because of its value for the local community jaborandi is considered one of the key plants for post-mining re-vegetation. Unfortunately, when cultivated Pilocarpus gives low levels of pilocarpine. It is thought that one of the reasons for this is the lack of environmental stressors (e.g., metals or low soil pH) that boost pilocarpine production in the wild. We believe that the molecular understanding of pilocarpine synthesis would shed light on jaborandi adaptation to disturbed environments. In turn, this may result in income generation for local communities and the pharmaceutical industry, as well as environmental benefits arising from more effective conservation and restoration practices of the mining industry.

## HOW TO PROTECT AND RESTORE?

In 1992 during the Earth Summit in Rio de Janeiro, the Convention on Biological Diversity (CDS) was signed to help creating legislation for biodiversity protection. Mineral rich regions present a serious dilemma for the CDS (Gibson et al., 2010; Jacobi et al., 2011). On the one hand, they are home to unique ecosystems, often considered biodiversity hot spots. On the other hand the demand for metallic ores is growing rapidly and so is the number of mining permits, a trend that will continue in the future. Thus to sustain metalliferous ecosystems, while at the same time ensuring satisfactory returns from the mining activities compromise must be made. As long-term outcomes of any restoration efforts cannot be predicted (see below), it is indisputable that parts of canga must be protected; exactly how much is a subject of debate. In Brazil state protected reserves, which are exempted from mining rights, cover relatively small part of mineral regions. In MG 39 km^2^ of canga lies within borders of Serra do Rola Moca State Park (Jacobi et al., 2008). In Carajas, iron ore is locked in two mountain ranges called Serra Sul and Serra Nord located at the territory of Natural Reserve Park, Flona de Carajas. Mining already takes place in Serra Nord (**Figure 1I**), and a new mining complex in Serra Sul, S11D, is planed to start producing in 2016. Importantly, parts of S11D will be exempted from mining, as a result of heavily negotiated agreements between environmental agencies and the mining company. For instance, of the 187 iron-stone caves 152 will be fully protected (Vale Sustainability Report, 2012). Moreover protection belt between mountain lakes, do Violão and do Amendoim, and the mining operation is planned to protect lakes and surrounding canga outcrop (**Figures 1C,D**).

In mining areas where protection is not possible ecosystem restoration is a legal requirement. In Brazil to obtain or renew mining permits, companies need to submit area restoration plans to be implemented upon mine closure. These include a plethora of steps that preceded the closure and sometimes even mining itself (see below). Moreover actions mitigating ecosystem destruction during mining operation need to be included. For example, as part of the compromise with the environmental agencies, a number of technological solutions have been implemented in the S11D Carajas complex, such as, truckless, conveyor belt operated delivery of the iron ore from the mining site to the processing plant outside Flona de Carajas reserve (Vale Sustainability Report, 2012, 2013). It is noteworthy that no such or similar solutions where required from mining companies, when exploitation started 30 years ago in Serra Nord. Similarly, in the past, it was legally acceptable to rehabilitate exhausted mining areas with plant cover from any available plant species (see Griffith and Toy, 2001 for the early re-vegetation strategies of iron mines in IQ). Nowadays the aim is to restore ecosystems relying exclusively on native taxa.

The dependence of canga on the unique biotic components mentioned earlier, makes restoration a difficult task. Iron ore open air mining is aggressive. Ironstone outcrops and associated biota are striped, so the iron ore deposits can be accessed with the subsequent excavation reaching even 300 m in depth. The landscape left at the end of the mining operation has an erased geographical topography, no previous terrain structure, biotic, and abiotic components left. Hydrology can be seriously disturbed due to changes in water table and erosion (Castro et al., 2011a,b). Despite these considerable difficulties, principal restoration steps do not differ when compared with recovery of other post-mining areas, and will be listed keeping canga in mind. (1) As mentioned previously, restoration goals are set before the mining begins and long before it ends. (2) Careful ecosystem reviews (e.g., Jacobi and Carmo, 2011) are used as restoration guidelines. Species information is collected together with habitat information (e.g., soil composition; for canga see, e.g., Nunes, 2009; Messias et al., 2013) and used for ecological modeling (reviewed by Memmott, 2009; Stouffer et al., 2012) to delineate and visualize relations between organisms (for canga seem, e.g., Araújo et al., 2006; Jacobi et al., 2008; Jacobi and Carmo, 2011). Based on the above, priority species and interactions are selected. For instance, detailed analysis of the angiosperm composition identified the following species as priority for canga recovery in IQ (1) grass *Andropogon ingratus* small shrub *Lychnospora pinaster,* and sedge *Bulbostylis fimbriata* as most abundant in terms of individual number and total area occupied (2) *Mimosa calodendron* as nitrogen fixing nursery taxa (3) Vellozia species for their metal tolerance and accumulation mechanisms (see previous section on metallophytes; Jacobi et al., 2008). Plants used by indigenous communities such as *Pilocarpus microphyllus* and endemic species such as *Arthrocereus glazowii* have been also considered as a priority. (3) Mining areas are prepared so as to minimize deforestation, e.g., as in the S11D complex by shifting all the ore processing and tailings outside Flona de Carajas territory. (4) Topsoil removal and storage is now being practiced, as it contains seed banks and associated micro-biomes, and was proven to be top priority for ecosystem restoration (Cooke and Johnson, 2002). For instance, work of Rezende et al. (2013) demonstrated successful growth of 14 out of 15 tested canga herbaceous, shrubby, and woody species, over the period of 42 months, using topsoil salvaged from iron ore mining sites in MG. Another published example is a pilot study done for a new iron-ore mining complex in IQ, called Minas Rio (Mello et al., 2014). Flora rescue operations from canga areas to be mined salvaged 45 plant species belonging to different families and life forms that were subsequently introduced together with 5 cm of top-soil to a different location. After a period of approximately 3 years almost 2000 individuals representing 38 plant species were recorded. Noteworthy, the majority of the species, such as the already mentioned sedge *Bulbostylis fimbriata, were* successfully reproduced within the experiment duration. Since 2010, a further 108 plant species have been rescued by the Minas Rio operation. (5) Landscape architecture to stabilize slopes and restore some of the original topography is also required to recreate functional ecosystem, as for example in case of Germano mine in MG (see below; Castro et al., 2011a) (6) Re-vegetation using available planting material. As described above, many canga species can be successfully rescued before mining begins and subsequently cultivated *ex situ* awaiting restoration. Seeds and plantings obtained from *ex situ* collections constitute the obvious starting material for re-vegetation. (7) During the early phases of restoration applying fertilizers and using chemical means to deal with invading plant species and a plethora of pests is often required. Introducing native legumes to fix nitrogen is not uncommon. (8) Monitoring criteria, which are essential to assess restoration progress, are usually decided at the early operation stages (Cooke and Johnson, 2002). Soil organic matter content and species richness are traditionally used, physiological parameters (Cooke and Suski, 2008), nowadays more and more accessible from the aerial photographs (e.g., ground cover, rate of biomass increase, chlorophyll content), are, however, increasingly more popular.

An often cited example of a successful restoration effort is the Jarrah forest in Western Australia (reviewed by Grant and Koch, 2007). Within 50 years of the mining operation closure the original ecosystem, with more than 700 native plant and 200 animal species, has been reproduced and nowadays is considered fully self-sustainable. It demonstrates that restoration is possible, where all involved parties commit to their obligations, main fear of the environmentalists. In Brazil, two examples of recently closed iron ore mines are Aguas Claras and Germano in MG (Castro et al., 2011a). Due to its location, area of Aguas Claras will be used to build a small town neighboring a new lake created in the depleted mining pit. In contrast, the Germano pit closure focuses on environmental rehabilitation; extensive landscape architecture being carried on at the moment. Closure time for the Minas Rio (MG) and S11D (Carajas), new projects that include canga restoration planing, is envisaged only in 30–40 years, respectively. As it is very long time before it will be possible to evaluate restoration success, and as mentioned before, the need of canga protection remains indisputable.

## SUMMARY, CANGA, AND MEANING OF BIODIVERSITY IN THE NEW MILLENNIUM

Although very small in size canga demonstrates biodiversity challenges of the ongoing Millenium. Due to its unique setting, canga significantly contributes to regional and global biodiversity. And as such is seen as a holy grail by conservationists and a treasure box by biologists. Laying on mineral deposits canga is, however, threatened by human activities, presenting an important dilemma between biodiversity and economical gains, ever more acute in the current millennium, with the human population and its prosperity rapidly growing. The difficult comprise between human economic activities and biodiversity is simply unavoidable. Protect and restore discussions are not easy and new concepts, such as environmental offsets, are emerging. Hopefully the current Millennium will bring new solutions to biodiversity preservation as it brings challenges. “The future is unknown but it can be invented.”

## Conflict of Interest Statement

The authors declare that the research was conducted in the absence of any commercial or financial relationships that could be construed as a potential conflict of interest. ITV-DS Institute is funded by mining company Vale, which has mining activities in the regions of canga occurence. This review is related to one of the ITV roles, which is support of canga characterization and restoration affords. Considering Frontiers is a scientific journal all authors kept to the objectivity criteria.

## References

[B1] Abreu V. G. Correa G. M. Silva T. M. Fontoura H. S. Cara D. C. Piló-Veloso D. (2013). Anti-inflammatory effects in muscle injury by transdermal application of gel with *Lychnophora pinaster* aerial parts using phonophoresis in rats. *BMC Complement Altern. Med.* 13:270 10.1186/1472-6882-13-270 PMC3874755 24138803

[B2] Araújo V. A. Antonini Y. Araújo A. P. (2006). Diversity of bees and their floral resources at altitudinal areas in the Southern Espinhaço Range, Minas Gerais, Brazil. *Neotrop. Entomol.* 35 30–40 10.1590/S1519-566X2006000100005 17352066

[B3] Baker A. J. M. (1981). Accumulators and excluders - strategies in response of plant to heavy metals. *J. Plant Nutr.* 3 643–654 10.1080/01904168109362867

[B4] Barbosa F. L. Mori L. S. Riva D. Stefanello M. É. Zampronio A. R. (2013). Antinociceptive and anti-inflammatory activities of the ethanolic extract, fractions and 8-methoxylapachenol from *Sinningia allagophylla*. *Basic Clin. Pharmacol. Toxicol.* 113 1–7 10.1111/bcpt.12051 23336113

[B5] Boyd R. S. (2012). Plant defense using toxic inorganic ions: conceptual models of the defensive enhancement and joint effects hypotheses. *Plant Sci.* 195 88–95 10.1016/j.plantsci.2012.06.012 22921002

[B6] Castro M. Lima H. Flores J. (2011a). Overview of mine closure in Minas Gerais, Brazil. *Rev. Esc. Minas Ouro Preto* 64 205–211 10.1590/S0370-44672011000200012

[B7] Castro P. Nalini H. Lima H. (2011b). *Understanding Mining around the Quadrilatero Ferrifero. First Edition*. Belo Horizonte: Ecológico Consultoria Ambiental.

[B8] Costa F. (2012). *Os folgeiros do Jaborandi: Organizacao, Parerias e seu Lugar No Extravitismo Amazonico*. *Universidade Federal de Para, Belém* 184.

[B9] Cooke J. A. Johnson M. S. (2002). Ecological restoration of land with particular reference to the monitoring of metals and industrial minerals: a review of theory and practice. *Environ. Rev.* 10 41–71 10.1139/a01-014

[B10] Cooke J. A. Suski C. D. (2008). Ecological restoration and physiology: an overdue integration. *Bioscience* 58 957–968 10.1641/B581009

[B11] Dorr J. N. (1964). Supergene iron ores of Minas Gerais, Brazil. *Econ. Geol.* 59 1203–1240 10.2113/gsecongeo.59.7.1203

[B12] de Melo J. O. Bitencourt T. A. Fachin A. L. Cruz E. M. de Jesus H. C. Alves P. B. (2013). Antidermatophytic and antileishmanial activities of essential oils from *Lippia gracilis* Schauer genotypes. *Acta Trop.* 128 110–115 10.1016/j.actatropica.2013.06.024 23850505

[B13] Erskine P. van der Ent A. Fletcher A. (2013). Sustaining metal-loving plants in mining regions. *Science* 337 1172–1173 10.1126/science.337.6099.1172-b 22955816

[B14] Fernando D. R. Marshall A. Baker A. J. Mizuno T. (2013). Microbeam methodologies as powerful tools in manganese hyperaccumulation research: present status and future directions. *Front Plant Sci.* 4:319 10.3389/fpls.2013.00319 PMC3747628 23970891

[B15] Ferraz R. P. Bomfim D. S. Carvalho N. C. Soares M. B. da Silva T. B. Machado W. J. (2013). Cytotoxic effect of leaf essential oil of *Lippia gracilis* Schauer (Verbenaceae). *Phytomedicine* 15 615–621 10.1016/j.phymed.2013.01.015 23453306

[B16] Forzza R. C. (2010). *Catálogo das Plantas e Fungos do Brasil* Vol. 2. Rio de Janeiro: Andrea Jakobsson Estúdio and Rio de Janeiro Botanical Garden.

[B17] Frankham B. (2010). Where are we in conservation genetics and where do we need to go? *Conserv. Genet.* 11 661–663 10.1007/s10592-009-0010-2

[B18] Gibson N. Meissner R. Markey A. S. Thompson W. A. (2012). Patterns of plant diversity in ironstone ranges in arid south Western Australia. *J. Arid Environ.* 77 25–31 10.1016/j.jaridenv.2011.08.021

[B19] Gibson N. Yates C. J. Dillon R. (2010). Plant communities of the ironstone ranges of south-western Australia: hotspots for plant diversity and mineral deposits. *Biodivers. Conserv.* 19 3951–3962 10.1007/s10531-010-9939-1

[B20] Grant C. Koch J. (2007). Decommissioning Western Australia’s first bauxite mine: co-evolving vegetation restoration techniques and targets. *Ecol. Manag. Restor.* 8 92–105 10.1111/j.1442-8903.2007.00346.x

[B21] Griffith J. J. Toy T. J. (2001). Evolution in revegetation of iron-ore mines in Minas Gerais State, Brazil. *Unasylva* 52 9–15.

[B22] Guzzo L. S. Saúde-Guimarães D. A. Silva A. C. Lombardi J. A. Guimarães H. N. Grabe-Guimarães A. (2008). Antinociceptive and anti-inflammatory activities of ethanolic extracts of *Lychnophora species*. *J. Ethnopharmacol.* 116 120–124 10.1016/j.jep.2007.11.006 18155374

[B23] Hale K. L. McGrath S. P. Lombi E. Stack S. M. Terry N. Pickering I. J. (2001). Molybdenum sequestration in *Brassica species.* A role for anthocyanins? *Plant Physiol.* 126 1391–1402 10.1104/pp.126.4.1391 11500539 PMC117140

[B24] Jacobi C. M. Carmo F. F. (2008a). The contribution of ironstone outcrops to plant diversity in the iron quadrangle, a threatened Brazilian landscape. *Ambio* 37 324–326 10.1579/0044-7447(2008)37[324:TCOIOT]2.0.CO;2 18686515

[B25] Jacobi C. M. Carmo F. F. (2008b). Diversidade dos campos rupestres ferruginosos no Quadrilátero Ferrífero, MG. *Megadiversidade* 4 25–32.

[B26] Jacobi C. M. Carmo F. F. (2011). Life-forms, pollination and seed dispersal syndromes in plant communities on ironstone outcrops, SE Brazil. *Acta Botanica Brasílica* 25 395–412 10.1590/S0102-33062011000200016

[B27] Jacobi C. M. Carmo F. F. Campos I. C. (2011). Soaring extinction threats to endemic plants in Brazilian metal-rich regions. *Ambio* 40 540–543 10.1007/s13280-011-0151-7 21848142 PMC3357811

[B28] Jacobi C. M. Carmo F. F. de Castro V. R. (2008). Estudo fitossociológico de uma comunidade vegetal sobre canga como subsídio para a reabilitação de áreas mineradas no quadrilátero ferrífero, MG. *Revista Árvore* 32 345–353 10.1590/S0100-67622008000200017

[B29] Jacobi C. M. Carmo F. F. Vincent R. C. Stehmann J. R. (2007). Plant communities on the ironstone outcrops - a diverse and endangered Brazilian ecosystem. *Biodivers. Conserv.* 16 2185–2200 10.1007/s10531-007-9156-8

[B30] Krämer U. (2010). Metal hyperaccumulation in plants. *Annu. Rev. Plant Biol.* 61 517–534 10.1146/annurev-arplant-042809-112158 20192749

[B31] Langfield R. D. Scarano F. J. Heitzman M. E. Kondo M. Hammond G. B. Neto C. C. (2004). Use of a modified microplate bioassay method to investigate antibacterial activity in the Peruvian medicinal plant *Peperomia galioides*. *J. Ethnopharmacol.* 94 279–281 10.1016/j.jep.2004.06.013 15325731

[B32] Leite T. C. de Sena A. R. Dos Santos Silva T. R. Dos Santos A. K. Uetanabaro A. P. Branco A. (2012). Antimicrobial activity of *Marcetia* DC species (Melastomataceae) and analysis of its flavonoids by reverse phase-high performance liquid chromatography coupled-diode array detector. *Pharmacogn. Mag.* 8 209–214 10.4103/0973-1296.99286 23060695 PMC3466456

[B33] Leitenmaier B. Küpper H. (2013). Compartmentation and complexation of metals in hyperaccumulator plants. *Front Plant Sci.* 4:374 10.3389/fpls.2013.00374 PMC3778397 24065978

[B34] Leporatti M. L. Pintore G. Foddai M. Chessa M. Piana A. Petretto G. L. (2014). Chemical, biological, morphoanatomical and antimicrobial study of *Ocotea puchury-major* Mart. *Nat. Prod. Res.* 28 294–300 10.1080/14786419.2013.858338 24274027

[B35] Lynch J. P. St Clair S. B. (2004). Mineral stress: the missing link in understanding how global climate change will affect plant in the real world soils. *Filed Crop Res.* 90 101–115 10.1016/j.fcr.2004.07.008

[B36] Markey A. S. Dillon S. J. (2010). Flora and vegetation of the banded iron formations of the Yilgarn Craton: the Booylgoo Range. *Conserv. Sci. W. Aust.* 7 503–529.

[B37] Marschner P. (2012). *Marschner’s mineral Nutrion of Hugher Plants*. Boston, MA: Academic Press.

[B38] Mello L. Camargo R. Medeiros D. Almeida R. Vasconcelos R. Toledo F. (2014). “Partnerships and early planning with good science: the key to long-term ecological and socio-economic success,” in *Proceedings of Mine Closure Solutions* (Ouro Preto: InfoMine) 26–30.

[B39] Memmott J. (2009). Food webs: a ladder for picking strawberries or a practical tool for practical problems? *Phil. Trans. R. Soc. Bot.* 364 1693–1699 10.1098/rstb.2008.0255 PMC2685425 19451120

[B40] Messias M. Leite M. Neto J. Kozovits A. Tavares R. (2013). Soil-vegetation relationship in quartzitic and ferruginous Brazilian rocky outcrops. *Folia Geobot.* 48 509–521 10.1007/s12224-013-9154-4

[B41] Moraes T. M. de Araújo M. H. Bernardes N. R. de Oliveira D. B. Lasunskaia E. B. Muzitano M. F. (2011). Antimycobacterial activity and alkaloid prospection of *Psychotria species* (Rubiaceae) from the Brazilian Atlantic Rainforest. *Planta Med.* 77 964–970 10.1055/s-0030-1250656 21243585

[B42] Morrissey J. Guerinot M. L. (2009). Iron uptake and transport in plants: the good, the bad, and the ionome. *Chem. Rev.* 109 4553–4567 10.1021/cr900112r 19754138 PMC2764373

[B43] Nistelberger H. Byrne M. Coates D. Roberts J. D. (2014). Strong phylogeographic structure in a millipede indicates pleistocene vicariance between populations on banded iron formations in semi-arid Australia. *PLoS ONE* 9:e93038 10.1371/journal.pone.0093038 PMC3963978 24663390

[B44] Novaes P. Imatomi M. Varela R. M. Molinillo J. M. Lacret R. Gualtieri S. C. (2013). Allelopathic potential of *Rapanea umbellata* leaf extracts. *Chem. Biodivers.* 10 1539–1548 10.1002/cbdv.201200367 23939802

[B45] Nunes J. (2009). *Floristica, Estrutura e Relacoes Solo-Vegetacau em Gradiente Fitofisionomico Sobre Canga, na Serra sul, Flona de Carajas*. *Universidade Federal de Viçosa, Viçosa* 101.

[B46] Oliveira S. H. F. Negreiros D. Fernandes G. W. Brabosa N. P. U. Rocha R. Almeida-Cortez J. C. (2009). Seedling growth of the invader *Calotropis procera* in ironestone rupestrian field and seasonally dry forest soils. *Neotrop. Biol. Conserv.* 4 69–76 10.4013/nbc.2009.42.01

[B47] Pifano D. S. Valente A. S. de Souza Almeida H. Alves de Melo P. H. Montianeli de Castro R. van den Berg E. (2010). Caracterização florística e fitofisionômica da Serra do Condado, Minas Gerais, Brasil. *Biota Neotrop* 10 55–71 10.1590/S1676-06032010000100005

[B48] Porto M. L. da Silva M. F. F. (1989). Tipos de vegetacoa metalofila em areas de Serra de Carajas e de Minais Gerais, Brazil. *Acta. Bot. Bras.* 3 13–21 10.1590/S0102-33061989000200002

[B49] Pradeep B. V. Tejaswini M. Nishal P. Pardhu G. Shylaja S. Kumar K. Ch. (2013). Phytochemical screening and antimicrobial activities of plant extract of *Lantana camara*. *J. Environ. Biol.* 34 645–649. 24617153

[B50] Reganold J. P. Andrews P. K. Reeve J. R. Carpenter-Boggs L. Schadt C. W. Alldredge J. R. (2010). Fruit and soil quality of organic and conventional strawberry agroecosystems. *PLoS ONE* 5:e12346 10.1371/journal.pone.0012346 PMC2931688 20824185

[B51] Rezende L. A. L. Dias L. E. Assis I. R. Braga R. (2013). Rehabilitation of ironestones outcrops degraded by mining activity in Minas Gerais state of Brazil. *J. Am. Soc. Mining and Reclamation* 2 151–159.

[B52] Ribeiro A. Piló-Veloso D. Romanha A. J. Zani C. L. (1997). Trypanocidal flavonoids from *Trixis vauthieri*. *J. Nat. Prod.* 60 836–841 10.1021/np970196p 9287419

[B53] Sarria A. L. Soares M. S. Matos A. P. Fernandes J. B. Vieira P. C. da Silva M. F. (2011). Effect of triterpenoids and limonoids isolated from *Cabralea canjerana* and *Carapa guianensis* (Meliaceae) against *Spodoptera frugiperda* (J. E. Smith). *Z. Naturforsch. C* 66 245–250 10.5560/ZNC.2011.66c0245 21812341

[B54] Silva M. F. F. (1992). Distribuicao de metais pesados na vegetacoa metalofila de Crajas. *Act. Bot. Bras.* 6 107–121.

[B55] Silva W. (2013). *Gradiente Vegetacional e Pedológico em Complexo Rupestre de Quartzito no Quadrilátero Ferrífero, Minas Gerais, Brazil*. *University Federal of Viçosa, Viçosa* 79.

[B56] Silvério M. S. Del-Vechio-Vieira G. Pinto M. A. Alves M. S. Sousa O. V. (2013). Chemical composition and biological activities of essential oils of *Eremanthus erythropappus* (DC) McLeisch (Asteraceae). *Molecules.* 18 9785–9796 10.3390/molecules18089785 23959191 PMC6270547

[B57] Skirycz A. Jozefczuk S. Stobiecki M. Muth D. Zanor M. I. Witt I. (2007). Transcription factor AtDOF4;2 affects phenylpropanoid metabolism in *Arabidopsis thaliana*. *New Phytol.* 175 425–438 10.1111/j.1469-8137.2007.02129.x 17635218

[B58] Skirycz A. Vandenbroucke K. Clauw P. Maleux K. De Meyer B. Dhondt S. (2011). Survival and growth of *Arabidopsis* plants given limited water are not equal. *Nat. Biotechnol.* 29 212–214 10.1038/nbt.1800 21390020

[B59] Soberón J. R. Sgariglia M. A. Sampietro D. A. Quiroga E. N. Sierra M. G. Vattuone M. A. (2010). Purification and identification of antibacterial phenolics from *Tripodanthus acutifolius* leaves. *J. Appl. Microbiol.* 108:1757–1768 10.1111/j.1365-2672.2009.04579.x 19922598

[B60] Souza A. B. Martins C. H. Souza M. G. Furtado N. A. Heleno V. C. de Sousa J. P. (2011). Antimicrobial activity of terpenoids from *Copaifera langsdorffii* Desf. against cariogenic bacteria. *Phytother. Res.* 25 215–220 10.1002/ptr.3244 20632306

[B61] Stouffer D. B. Sales-Pardo M. Sirer M. I. Bascompte J. (2012). Evolutionary conservation of species roles in food webs. *Science* 335 1489–1492 10.1126/science.1216556 22442483

[B62] Taba P. (2013). Metals and movement disorders. *Curr. Opin. Neurol.* 26 435–441 10.1097/WCO.0b013e3283629beb 23787769

[B63] Vale Sustainability Report. (2012). Available at: http://www.vale.com/EN/aboutvale/sustainability/links/LinksDownloadsDocuments/2012-sustainability-report.pdf

[B64] Vale Sustainability Report. (2013). Available at: http://www.vale.com/EN/aboutvale/sustainability/links/LinksDownloadsDocuments/2013-sustainability-report.pdf

[B65] van der Ent A. Baker A. J. M. Reeves R. D. Pollard A. J. Schat H. (2013). Hyperaccumulator of metals and metaloid traces: facts and fiction. *Plant Soil* 362 319–334 10.1007/s11104-012-1287-3

[B66] Viana P. L. Lombardi J. A. (2007). Florística e caracterização dos campos rupestres sobre canga na Serra de Calcada, Minais Gerais, Brazil. *Rodriguesia* 58 159–177.

[B67] Vincent R. C. Meguro M. (2008). Plant soil relationships in ferruginous rocky soil vegetation. *Revista Brazil. Bot.* 31 377–388.

[B68] Whiting S. N. Reeves R. D. Richards D. Johnson M. S. Cooke J. A. Malaisse F. (2004). Research priorities for conservation of metallophyte biodiversity and their potential for restoration and site remediation. *Restor. Ecol.* 12 106–116 10.1111/j.1061-2971.2004.00367.x

[B69] Zimmermam-Franco D. C. Bolutari E. B. Polonini H. C. do Carmo A. M. Chaves M. D. Raposo N. R. (2013). Antifungal activity of *Copaifera langsdorffii* desf oleoresin against dermatophytes. *Molecules* 18 12561–12570 10.3390/molecules181012561 24126374 PMC6270220

